# Effects of Exposure Duration and Exposure Levels of Ambient Air Pollutants on the Risk of Polycystic Ovarian Syndrome: A 2015–2019 Korean Population-Based Cohort Study

**DOI:** 10.3390/toxics10090542

**Published:** 2022-09-18

**Authors:** Ju-Hee Kim, Se-Hwa Hong, Na-Lae Moon, Dae-Ryong Kang

**Affiliations:** 1 Department of Nursing, College of Nursing Science, Kyung Hee University, Seoul 02447, Korea; 2 Department of Biostatistics, Wonju College of Medicine, Yonsei University, Wonju 26426, Korea; 3 Department of Precision Medicine, Wonju College of Medicine, Yonsei University, Wonju 26426, Korea

**Keywords:** polycystic ovarian syndrome, ambient air pollution, exposure duration, exposure level, population-based cohort study

## Abstract

Exposure to ambient air pollution is associated with an increased risk of menstrual disorders and infertility. This study examined the relationships between the levels and duration of air pollution exposure and the risk of polycystic ovarian syndrome (PCOS) using Korean population-based cohort data (2015–2019). Real-time data on PM_10_, PM_2.5_, O_3_, CO, SO_2_, and NO_2_ were provided by the Korean Ministry of Environment. The average monthly air pollutant concentration from 1 January 2014 to 31 December 2018 was analyzed. To assess individual-level exposure to air pollutants, a spatial prediction model and an area-averaging approach were used. In total, 237,582 PCOS cases were analyzed. The annual age-adjusted PCOS incidence was 6.70, 8.28, 9.73, 11.58, and 11.97% from 2015–2019, respectively. The PCOS risk increased 1.29–1.32, 1.43–1.52, and 1.32-fold following exposure to the 2-year and 3-year average levels of PM_2.5_, O_3_, and NO_2_, respectively, compared to their 1-year average levels. The PCOS risk increased 1.75-fold (95% confidence interval: 1.66–1.85) in the fourth-quartile for the NO_2_ level. Increased SO_2_ and CO levels in the second- and third-quartiles were also associated with an increased PCOS risk. Exposure to air pollutants thus increased the risk for PCOS in the Korean population.

## 1. Introduction

Many epidemiological studies and review papers have reported associations between air pollutant exposure and poor reproductive health, including hormonal abnormalities [1,2], menstrual disorders [3,4,5,6], infertility [5,6,7,8,9], gynecological disorders [6,10], and adverse perinatal outcomes [11,12,13,14]. Although the mechanisms whereby air pollutant exposure contributes to poor reproductive health have not been definitively established, inflammation, oxidative stress, hypothalamus–pituitary–adrenal (HPA) axis activation, and DNA damage are thought to be involved [8,15,16,17,18]. One animal study reported that O_3_ exposure induced inflammation and pulmonary injury by increasing the level of stress hormones [19]. Exposure to PM_2.5_ activates the HPA axis and affects the follicle-stimulating hormone, the luteinizing hormone, and testosterone, which negatively influences ovum and sperm development and ultimately leads to infertility [20,21,22,23]. Air pollutants also induce oxidative stress, decrease plasma reproductive hormone levels [24,25,26], induce ovarian and testicular apoptosis [27], and disrupt DNA methylation [28,29].

Polycystic ovarian syndrome (PCOS) is a common endocrine disorder characterized by menstrual dysfunction, anovulation, hirsutism, hyperandrogenism, hypersecretion of the luteinizing hormone, and multiple ovarian cysts [30,31,32]. The worldwide prevalence of PCOS in women of reproductive age is 2.2–26.0% [33,34]. Although the etiology of PCOS is multi-factorial, recent environmental studies have emphasized that environmental toxicants contribute to increased androgen levels, anovulation, and the development of PCOS [17]. González [35] reported that pro-inflammatory stimuli can contribute to the development of PCOS by increasing the level of ovarian steroid enzymes, while Lin et al. [36] revealed that air pollution induces an excess of androgens through insulin resistance, eventually leading to PCOS.

Although a link between air pollution and poor reproductive health has been reported, to date, only two studies have investigated the association between air pollution and PCOS, with inconsistent results. On the one hand, Lin et al. [36] studied the effect of air pollution on the risk of developing PCOS among 91,803 Chinese women from 2000 to 2013 and found a 3.56–10.31 increase in the risk of PCOS. On the other hand, Fruh et al. [37] found no association between PM_2.5_ exposure and polycystic ovarian morphology in 5492 American women. In general, it takes a long time for a disease to develop following exposure to environmental harmful factors; thus, relevant studies must take the effect of time into account [38,39]. To accurately analyze the effects of air pollutants on PCOS risk, the sample size should be large, and possible confounding factors should be appropriately controlled for using an appropriate cohort design [40].

Therefore, this study analyzed the effects of the exposure duration and the levels of ambient air pollutants on PCOS risk using data from a Korean nationwide population-based cohort. To the best of our knowledge, this is the first study to examine the association between the duration of exposure to air pollutants and the risk of PCOS.

## 2. Materials and Methods

### 2.1. Study Design

**Figure 1:**
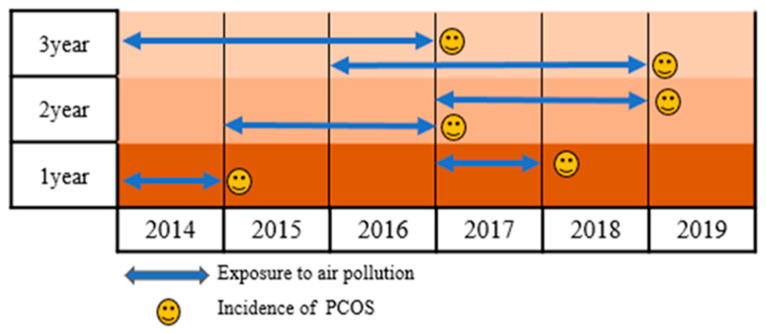
Study timeline.

This retrospective cohort study analyzed Korean nationwide population-based data from 1 January 2015 to 31 December 2019. The exposure duration included 1-year, 2-year, and 3-year data collected from 1 January 2014 to 31 December 2018 (Figure 1).

### 2.2. Study Source and Participants

**Figure 2:**
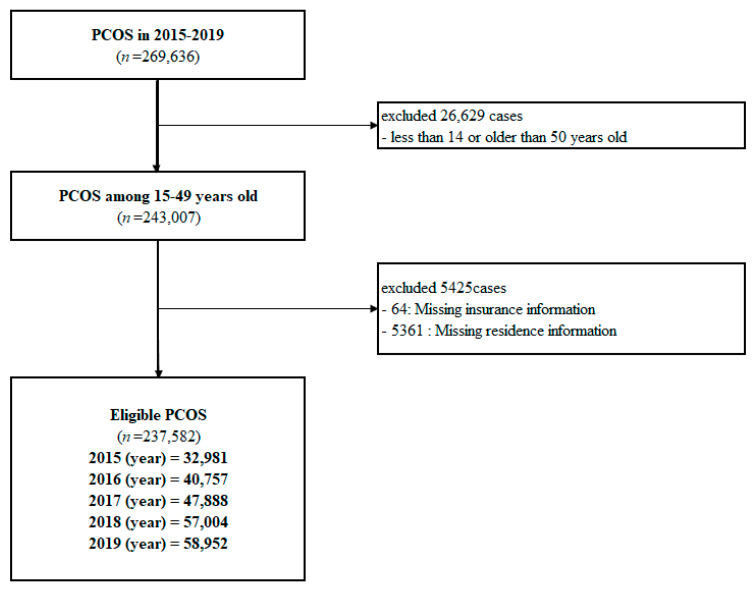
Flow chart of participant inclusion.

The National Health Information Database (NHID) is a nationwide database with population-based cohort data managed by the Korean National Health Insurance Service (KNHIS), which provides comprehensive medical services to all Korean citizens. The NHID includes personal demographic information, medical treatment, insurance data according to employment, and medical aid beneficiaries. We used the PCOS data from the NHID between 2015 and 2019 according to the following inclusion and exclusion criteria. Women meeting the following criteria were included: (1) 15–49-year-old; (2) Korean Informative Classification of Disease, 10th revision: E28.0–E28.9; and (3) newly diagnosed with PCOS. We excluded women with a second diagnosis of PCOS (cases of overlapping diagnosis) or with missing information regarding residence and insurance. From 2015 to 2019, we searched the PCOS data on the KNHIS-NHID system. Among the 269,636 women who were diagnosed with PCOS, 32,054 did not meet the exclusion criteria (Figure 2). The final sample included 237,582 cases. This study was approved by the Institutional Review Board of the Wonju Severance Christian Hospital (approval number CR321311).

### 2.3. Exposure Assessment

The Ministry of Environment of Korea provides nationwide real-time data on outdoor air pollutants, such as PM_10_, PM_2.5_, O_3_, CO, SO_2_, and NO_2_, every hour on the Air Korea (www.airkorea.or.kr, accessed on 7 March 2022) website. The ground air pollution monitoring data are measured at 355 air monitoring stations in 17 provinces and 254 cities nationwide. We used a spatial prediction model and area-averaging approach to assess individual-level exposure to air pollutants. The spatial prediction model included data on spatial correlation with predictors of more than 300 geographic variables, including land usage information, population demographic information, and emissions [11]. The concentration of air pollution was estimated using administrative data based on individual addresses on KNHIS-NHID. We used the monthly average concentrations of air pollutants (PM_10_, O_3_, CO, SO_2_, and NO_2_) from 2014 to 2018. Since PM_2.5_ data are provided from 2015 in Korea, the risk effect was calculated from 2016. Subsequently, the residence (county, city, and province) of the participants was matched with the concentration of air pollutants. We used the 1-year-, 2-year-, and 3 year-average monthly air pollutant concentration. “Exposure for 1 year” was defined as the level of exposure to air pollution among individual women diagnosed with PCOS over the past year. Similarly, “exposure for 2 years” and “exposure for 3 years” were defined as the level of exposure to air pollution among individual women diagnosed with PCOS over the past two and three years, respectively. Additionally, the concentrations of PM_10_, PM_2.5_, SO_2_, CO, O_3_, and NO_2_ were divided according to quartiles.

### 2.4. Statistical Analysis

We used SAS version 9.4 (SAS Institute Inc., Cary, NC, USA) to conduct the statistical analyses. The R software was used to visualize the data on the concentration of air pollutants. The age-adjusted annual incidence rates of PCOS from 2010 to 2019 were calculated by dividing the number of women diagnosed with PCOS by the number of Korean women from the 2010 Population and Housing Census. The age-adjusted incidence rate was calculated by dividing the number of annual new cases of PCOS by the number of women at risk. The number of women at risk for each year was calculated using the following equation: [total number of Korean women from the 2010 Population and Housing Census—(number of pre-existing cases in the previous year + half of the number of new cases in the year)].

PM_2.5_ and PM_10_ were expressed in μg/m^3^, and NO_2_, O_3_, CO, and SO_2_ were expressed in ppb. Air Korea provides data in ppm; thus, we converted the data to ppb (by multiplication by 1000) for comparison with previous studies. We used logistic regression analysis to analyze the effect of the exposure duration and levels of air pollutants on PCOS risk and calculated the OR and 95% confidence intervals. For individual covariates, we included age (<35 years old vs. ≥35 years old), residence (urban and rural), household income (low, middle, and high), body mass index (kg/m^2^), high-density lipoprotein (HDL) cholesterol (mg/dL), and fasting blood glucose (mg/dL), based on a previous study [41]. We categorized the household income according to the insurance premiums in Korea. The income group was categorized into 20 classes: class 1 (lowest income) to class 20 (highest income). Hence, we categorized the income groups into low (class 1 to 6), middle (class 7 to 13), and high (class 14 to 20) groups. Continuous numerical data were described as the mean along with the standard deviation. A *p*-value < 0.05 was considered statistically significant.

## 3. Results

### 3.1. Annual Incidence and Characteristics of PCOS

**Figure 3:**
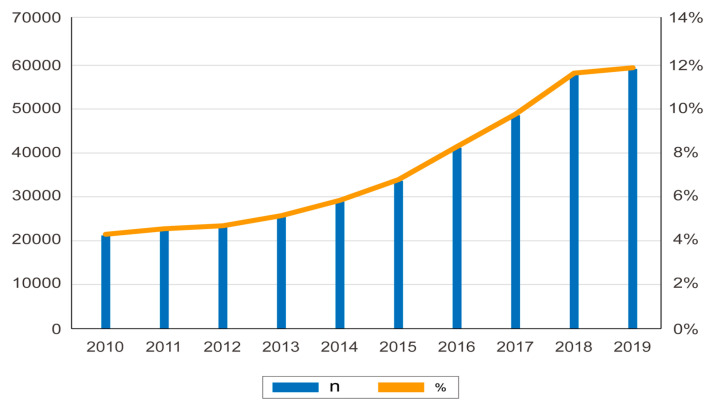
Annual incidence and prevalence of polycystic ovarian syndrome in Korea.

The age-adjusted incidence and prevalence of PCOS steadily increased annually from 2010 to 2019 (Figure 3) (Table S1). A total of 237,582 PCOS cases from 2015–2019 were included in this study. Overall, the age-adjusted incidence and prevalence of women with PCOS among the Korean population were 2.8% and 4.3% over the past 10 years (2010–2019), respectively [41]. 

The characteristics of the study population are presented in Table 1. The mean age of the study participants ranged from 30.68 (9.05) to 30.98 (8.97) years old. There were more PCOS cases in the group aged < 35 years than there were in the group aged ≥ 35 years. Regarding economic status, the low-income group had the highest number of PCOS cases, followed by the middle- and high-income groups. There were approximately three times as many PCOS cases in urban areas than there were in rural areas.

The income group was categorized into 20 classes: class 1 (lowest income) to class 20 (highest income). There were low (class 1 to 6), middle (class 7 to 13), and high (class 14 to 20) groups.

### 3.2. Concentration of Air Pollutants According to Exposure Duration

The concentrations of air pollutants according to the duration of exposure are described in Table 2. The mean concentrations of PM_10_, CO, and NO_2_ increased gradually from 1 year to 3 years. The maximum exposure concentration was the highest after 3 years for all air pollutants.

### 3.3. Effects of Exposure Duration of Air Pollutants on Polycystic Ovarian Syndrome Risk

**Figure 4:**
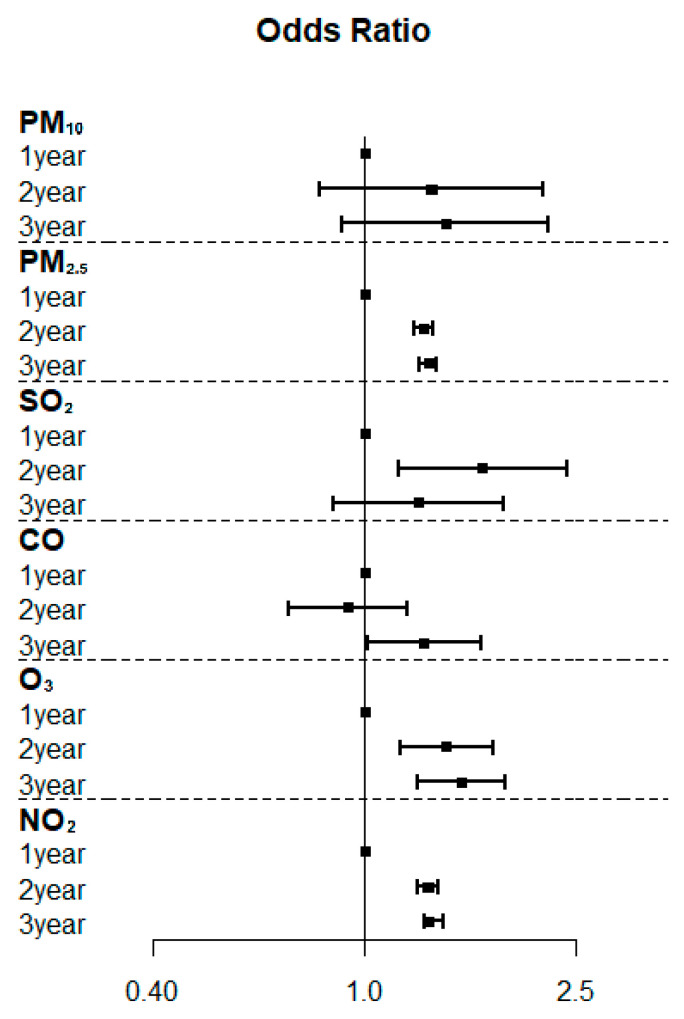
Forest plots of the association between the duration of exposure to air pollutants and the risk of polycystic ovarian syndrome.

Compared to the 1-year average concentration, the 2-year and 3-year average concentrations of PM_2.5_, O_3_, and NO_2_ significantly increased PCOS risk, with adjusted ORs of 1.29–1.32 (*p* < 0.001–0.03), 1.43–1.52 (*p* < 0.001), and 1.32 (*p* < 0.001), respectively (Table 3). The association between the duration of exposure to air pollutants and the risk of PCOS is illustrated in a forest plot (Figure 4).

### 3.4. Effects of the Exposure Concentration of Air Pollutants on the Risk for Polycystic Ovarian Syndrome

Table 4 summarizes the effects of air pollutant concentrations on PCOS risk. Further details are provided in Figure S1. PCOS risk was the highest at the fourth-quartile of the NO_2_ level (adjusted OR, 1.75; 95% CI: 1.66–1.85), and the risk of PCOS increased as the concentrations of NO_2_ increased. The levels of SO_2_ and CO were the highest in the third-quartile and were associated with an increased risk of developing PCOS (adjusted OR, 1.16–1.21 and 1.39–1, respectively). However, the risk of PCOS decreased as the concentrations of PM_2.5_ increased.

## 4. Discussion

This study showed that PCOS risk increases in parallel with the duration of exposure to PM_2.5_, O_3_, and NO_2,_ and the exposure concentration of SO_2_, CO, and NO_2_. To the best of our knowledge, this is the first study to examine the association between the duration of exposure to air pollutants and the risk of PCOS.

In this study, the incidence and prevalence of PCOS were 2.8 and 4.3%, which are similar to or slightly lower than those of previous studies [42,43,44,45,46]. These differences can be understood as differences in race, diagnostic criteria (NIH diagnosis and Rotterdam criteria, among others), and study design. Further research is needed to evaluate why the risk of PCOS gradually increases.

In this study, the PCOS risk increased 1.29–1.32 times as the duration of exposure to PM_2.5_ increased. These results are similar to those of previous studies showing the long-term adverse effects of air pollution on reproductive health [3,4,5,6,47]. Exposure to PM_2.5_ increases cardio-metabolic risk by stimulating the HPA axis and enhancing the secretion of the corticotropin-releasing hormone, the adrenocorticotropic hormone, and cortisol [8,20]. A possible mechanism underscoring this association is that most compounds constituting PM act as xenoestrogen and bind to estrogen receptors in target tissues [48,49]. Polycyclic aromatic hydrocarbons have a negative effect on follicular activity and increase the risk for infertility, reproductive dysfunction, and breast cancer [3,50]. Additionally, PM increases oxidative stress and inflammation. Oxidative stress changes the intracellular calcium level of oocytes, while antioxidants stimulate ovarian cyst formation [17,24,51]. However, in this study, the risk of PCOS was inconsistent with respect to the level of exposure to air pollutants. These results are similar to those of Fruh et al. [37], who reported a limited association between PM_2.5_ concentration and polycystic ovarian morphology. In contrast, Lin et al. [36] reported that, compared to the first-quartile levels, the third- and fourth-quartile levels of exposure to PM_2.5_ increased PCOS risk by 3.94 and 3.56 times, respectively. This difference is attributed to exposure duration and the measurement method of PM. Lin et al. [36] used the average daily air pollution concentration one year before PCOS diagnosis, whereas we used the monthly average concentration of air pollutants.

Moreover, we found that increased PCOS risk was associated with both a longer duration of exposure to NO_2_ (1.35-fold increase) and a higher concentration of NO_2_ (1.75-fold increase). These results are consistent with those of previous studies [1,4,6,36]. NO_2_ is a traffic-related air pollutant that is rapidly formed by the reaction between NO and O_3_ in the atmosphere [3,52]. NO_2_ exposure adversely affects reproductive health, depending on both exposure duration and concentration. Lin et al. [36] reported that, as the concentration of NO_X_, NO, and NO_2_ exposure increased, the risk of PCOS occurrence increased by 3.37, 4.18, and 7.46 times, respectively. A cohort study in China using a time-series analysis found that an increase of 10 μg/m^3^ in NO_2_ exposure increased menstrual disease by 2.17% [4]. In addition, NO_2_ exposure was positively correlated with estradiol and progesterone levels and was inversely correlated with anti-mullerian hormone (AMH) levels [1,2].

Furthermore, we found that the risk of PCOS increased as the concentration of SO_2_ increased, but the risk decreased at the highest concentration. These results are in line with those of previous studies [2,3]. SO_2_ exposure showed an inverse correlation with estradiol and progesterone concentrations and a positive correlation with the T/E2 (testosterone/estradiol) ratio [2]. A Polish study reported that exposure to SO_2_ shortened the luteal phase of the ovaries, resulting in irregular menstrual cycles [3]. Lin et al. [36] found that exposure to high concentrations of SO_2_ could increase the risk of PCOS by as much as 10.31 times. However, Liang et al. [4] observed no association between SO_2_ exposure and the incidence of menstrual disorders, and this discrepancy in results is presumed to be due to exposure duration. Whereas most studies used exposure concentrations of at least one year before disease diagnosis, Liang et al. [4] investigated the short-term effect in a week immediately before disease onset. Since SO_2_ is classified as a fossil fuel-related air pollutant [3], it is more appropriate to evaluate the risk of SO_2_ exposure to confirm the long-term rather than the short-term effects.

We also confirmed that the risk for PCOS increases according to the duration of exposure to O_3_ and the concentration of CO. Previous studies have reported inconsistent results. For example, Wang et al. [2] reported that CO exposure has a positive association with progesterone, whereas Merklinger-Gruchala et al. [3] reported no association between CO exposure and the overall ovarian cycle length. CO directly combines with hemoglobin to form carboxyhemoglobin, which is much more stable than oxyhemoglobin, thus preventing red blood cells from binding to oxygen. Since CO can negatively affect reproductive health through this mechanism, further studies are required in order to understand our results. Although information regarding the relationship of O_3_ exposure and reproductive health is lacking, two systematic reviews have reported that O_3_ exposure has a negative effect on the total sperm count and live birth rate [7,8]. In addition, considering that O_3_ reacts with NO to form NO_2_, the actual effect is thought to be greater [52].

This study has several limitations. First, since the exposure of air pollutants was based on the registered address of the study participant, if the actual address and the administrative address were different, the exposure measurement may not be accurate. Second, we used the 1-year, 2-year, and 3-year average pollutant concentration just before PCOS diagnosis to confirm the effect of exposure duration on PCOS risk. However, the average concentration does not reflect the cumulative effect of air pollutants; thus, future studies should address this issue using a time-lag model [38,39]. Third, PCOS is known to be associated with many socioeconomic and medical variables [53,54,55]. However, in this study, only age, residence, economic level, body mass index, HDL cholesterol, and FBS variables were used, because the cohort constructed by the Korean government for the entire population was analyzed. Nevertheless, this study was representative of the entire Korean population, given that we analyzed nationwide population-based cohort data and calculated the incidence of PCOS from 2015–2019 in Korea. In addition, this study was the first to evaluate the PCOS risk according to the exposure duration and the exposure concentration of air pollutants.

## 5. Conclusions

This study confirmed the association between increased PCOS risk and a greater duration and level of exposure to PM_10_, PM_2.5_, SO_2_, CO, O_3_, and NO_2_. Our results indicated that PCOS risk is associated with the exposure duration of PM_2.5_, O_3_, and NO_2_ and with the concentrations of SO_2_, CO, and NO_2_. Exposure to ambient air pollution during reproductive age adversely affects menstrual and ovarian disorders, such as PCOS, and may eventually lead to reduced fertility and infertility. Future studies should analyze not only outdoor air pollutants but also indoor air pollutants and construct a model to confirm the cumulative effect of the exposure duration of air pollutants.

## Figures and Tables

**Table 1:** Characteristics of women with polycystic ovarian syndrome included in this study (*n* = 237,582).

	Year				
	2015	2016	2017	2018	2019
Age, years					
*N*	32,981	40,757	47,888	57,004	58,952
Mean (SD)	30.76 (8.90)	30.75 (9.11)	30.68 (9.05)	30.87 (8.90)	30.98 (8.97)
Range, *n*					
<35 years old	22,609	27,638	32,637	38,035	39,417
≥35 years old	10,372	13,119	15,251	18,969	19,535
Income, *n*					
Low	12,935	15,993	18,758	22,594	22,870
Middle	12,041	14,551	17,050	20,412	21,115
High	8005	10,213	12,080	13,998	14,967
Residence, *n*					
Urban	25,126	31,292	37,012	43,800	45,153
Rural	9155	9465	10,876	13,204	13,799

**Table 2:** Concentration of air pollutants according to exposure duration.

Year	Pollutants	Mean	SD	Min	25th	50th	75th	Max
1 years	PM_10_	44.2	7.5	21.8	39.5	44.5	49.0	71.7
	PM_2.5_	25.5	3.5	14.3	23.3	25.3	27.5	39.2
	SO_2_	4.5	1.3	0.8	3.7	4.4	5.3	12.7
	CO	494.8	92.6	166.7	433.3	491.7	558.3	825.0
	O_3_	26.5	4.3	17.2	23.4	26.1	2.9	56.9
	NO_2_	23.9	7.1	1.9	18.6	24.0	30.0	39.9
2 years	PM_10_	44.6	6.5	27.4	39.8	44.6	49.1	64.7
	PM_2.5_	25.7	3.1	15.6	24.0	25.6	27.4	38.2
	SO_2_	4.5	1.2	1.3	3.7	4.5	5.2	12.7
	CO	498.1	86.7	179.2	445.8	495.8	554.2	825.0
	O_3_	26.6	4.1	18.6	23.8	26.1	29.0	56.9
	NO_2_	24.0	6.3	2.8	19.1	24.2	30.2	39.9
3 years	PM_10_	44.8	5.9	29.8	40.1	44.5	49.1	61.2
	PM_2.5_	25.7	2.8	16.1	23.9	25.5	27.5	36.1
	SO_2_	4.5	1.2	1.1	3.8	4.6	5.2	12.7
	CO	499.5	84.0	183.3	450.0	497.2	552.8	825.0
	O_3_	26.5	3.9	41.9	23.9	26.1	28.8	56.9
	NO_2_	24.1	6.9	3.2	109.1	24.3	30.3	39.9

**Table 3:** Association between exposure duration of air pollutants and PCOS.

Air Pollutants	Exposure Duration	Crude OR (95% CI)	*p*-Value	Adjusted OR (95% CI)	*p*-Value
PM_10_	2 years	1.11 (0.90–1.38)	0.334	1.34 (0.82–2.17)	0.241
	3 years	1.16 (0.94–1.42)	0.169	1.42 (0.90–2.22)	0.130
PM_2.5_	2 years	1.52 (1.21–1.91)	<0.001	1.29 (1.24–1.34)	0.032
	3 years	1.50 (1.20–1.88)	<0.001	1.32 (1.27–1.37)	0.030
SO_2_	2 years	1.28 (1.08–1.52)	0.005	1.67 (1.16–2.41)	0.011
	3 years	1.25 (1.06–1.48)	0.003	1.26 (0.87–1.82)	0.220
CO	2 years	1.20 (1.07–1.35)	<0.001	0.93 (0.72–1.20)	0.584
	3 years	1.38 (1.23–1.54)	<0.001	1.29 (1.01–1.65)	0.041
O_3_	2 years	1.17 (1.06–1.28)	<0.001	1.43 (1.17–1.74)	<0.001
	3 years	1.21 (1.11–1.32)	<0.001	1.52 (1.25–1.84)	<0.001
NO_2_	2 years	1.26 (1.23–1.28)	<0.001	1.32 (1.26–1.37)	<0.001
	3 years	1.28 (1.26–1.31)	<0.001	1.32 (1.29–1.41)	<0.001
Σ air pollutants	2 years	1.25 (1.23–1.28)	<0.001	1.31 (1.26–1.37)	<0.001
	3 years	1.28 (1.26–1.30)	<0.001	1.36 (1.30–1.41)	<0.001

Regression models were adjusted for age, residence, income, body mass index, high-density lipoprotein cholesterol, and fasting blood glucose. Reference values: Q1 in PM_10_, PM_2.5_, CO, NO_2_, and SO_2_. OR, odds ratio; CI, confidence interval.

**Table 4:** Association between exposure concentration and risk for polycystic ovarian syndrome.

		1 Year	*p*-Value	2 Years	*p*-Value	3 Years	*p*-Value
		Adjusted OR (95% CI)		Adjusted OR (95% CI)		Adjusted OR (95% CI)	
PM_10_	Q2	0.85 (0.82–0.88)	<0.001	0.83 (0.80–0.86)	<0.001	0.94 (0.89–0.98)	0.003
	Q3	0.96 (0.93–1.00)	0.004	0.97 (0.93–1.01)	0.127	0.97 (0.93–1.01)	0.158
	Q4	0.92 (0.89–0.96)	<0.001	0.92 (0.89–0.96)	<0.001	0.95 (0.90–0.99)	0.016
PM_2.5_	Q2	1.24 (1.20–1.28)	<0.001	1.22 (1.18–1.27)	<0.001	1.28 (1.22–134)	<0.001
	Q3	1.22 (1.18–1.27)	<0.001	1.13 (1.08–1.17)	0.998	1.06 (1.01–1.11)	<0.001
	Q4	1.16 (1.12–1–20)	<0.001	1.17 (1.12–1.21)	0.003	1.17 (1.12–123)	0.002
SO_2_	Q2	1.11 (1.07–1.15)	<0.001	1.12(1.07–1.16)	<0.001	1.18 (1.13–124)	<0.001
	Q3	1.16 (1.11–1.20)	<0.001	1.22 (1.17.1.27)	<0.001	1.21 (1.15–1.27)	<0.001
	Q4	0.99 (1.00–1.03)	0.672	0.99 (0.95–1.04)	0.747	1.01 (0.96–0.06)	0.696
CO	Q2	1.22 (1.17–1.26)	<0.001	1.27 (1.22–1.32)	<0.001	1.19 (1.14–125)	<0.001
	Q3	1.39 (1.34–1.44)	<0.001	1.53 (1.47–1.59)	<0.001	1.53 (1.46–1.60)	<0.001
	Q4	1.32 (1.28–1.37)	<0.001	1.38 (1.32–1.43)	<0.001	1.37 (1.31–1.43)	<0.001
O_3_	Q2	0.92 (0.89–0.96)	<0.001	0.88 (0.84–0.91)	<0.001	0.89 (0.85–0.93)	<0.001
	Q3	0.81 (0.78–0.84)	<0.001	0.73 (0.70–0.76)	<0.001	0.70 (0.67–0.73)	<0.001
	Q4	0.71 (0.68–0.74)	<0.001	0.62 (0.59–0.65)	<0.001	0.61 (0.59–0.65)	<0.001
NO_2_	Q2	1.00 (0.97–1.04)	0.923	0.98 (0.94–1.02)	0.304	1.02 (0.97–1.07)	0.411
	Q3	1.36 (1.31–1.42)	<0.001	1.30 (1.29–1.42)	<0.001	1.40 (1.33–1.48)	<0.001
	Q4	1.58 (1.51–1.35)	<0.001	1.66 (1.59–1.74)	<0.001	1.75 (1.66–1.85)	<0.001

Regression models were adjusted for age, residence, income, body mass index, high-density lipoprotein cholesterol, and fasting blood glucose. Reference values: Q1 in PM_10_, PM_2.5_, CO, NO_2_, and SO_2_. OR, odds ratio; CI, confidence interval.

## Supplementary Materials

The following supporting information can be downloaded at: https://www.mdpi.com/article/10.3390/toxics10090542/s1, Figure S1: Forest plot of the association between pollutant concentrations and polycystic ovarian syndrome; Table S1: Annual incidence of PCOS in Korea.
