# Supplementary material for: Effects of Exposure Duration and Exposure Levels of Ambient Air Pollutants on the Risk of Polycystic Ovarian Syndrome: A 2015–2019 Korean Population-Based Cohort Study
Source: Toxics. 2022 Sep 18;10(9):542. doi: 10.3390/toxics10090542 (PMC9501187; doi:10.3390/toxics10090542)
Supplement: Supplementary file 1 [file toxics-10-00542-s001.zip › toxics-1876112-supplementary.pdf]

Table S1. Annual incidence of PCOS in Korea.

Year	PCOS	
	n	%
2002	13,611	2.76
2003	14,669	2.98
2004	16,757	3.40
2005	17,201	3.49
2006	17,655	3.59
2007	17,915	3.64
2008	18,251	3.71
2009	19,217	3.90
2010	20,950	4.25
2011	22,121	4.49
2012	22,936	4.66
2013	25,084	5.09
2014	28,417	5.77
2015	32,981	6.70
2016	40,757	8.28
2017	47,888	9.73
2018	57,004	11.58
2019	58,952	11.97
Total	492,366	100.00

PCOS, polycystic ovarian syndrome

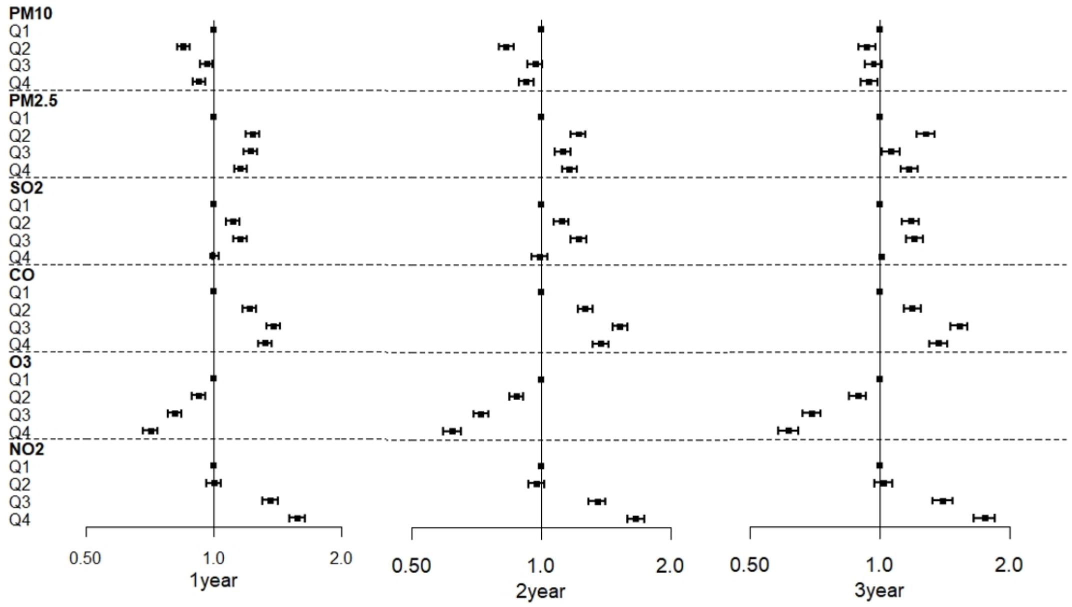

Figure S1. Forest plot of the association between pollutant concentrations and polycystic ovarian syndrome.
